# Inhibition of the Receptor for Advanced Glycation End Products Enhances the Cytotoxic Effect of Gemcitabine in Murine Pancreatic Tumors

**DOI:** 10.3390/biom11040526

**Published:** 2021-04-01

**Authors:** Priyanka Swami, Kelly A. O’Connell, Swetha Thiyagarajan, Ayrianne Crawford, Prathamesh Patil, Prakash Radhakrishnan, Simon Shin, Thomas C. Caffrey, James Grunkemeyer, Tammi Neville, Stefan W. Vetter, Michael A. Hollingsworth, Estelle Leclerc

**Affiliations:** 1 Department of Pharmaceutical Sciences, North Dakota State University, Fargo, ND 58015, USA; swamipriyanka17@gmail.com (P.S.); swetha.thiyagarajan@ndsu.edu (S.T.); Tammi.Neville@ndsu.edu (T.N.); Stefan.Vetter@ndsu.edu (S.W.V.); 2 Eppley Institute for Research in Cancer, University of Nebraska Medical Center, Omaha, NE 68198, USA; koconnell@unmc.edu (K.A.O.); Ayrianne.crawford@unmc.edu (A.C.); patilpprathamesh@gmail.com (P.P.); pradhakr@unmc.edu (P.R.); simon.shin@unmc.edu (S.S.); Thomas.Caffrey@unmc.edu (T.C.C.); James.Grunkemeyer@unmc.edu (J.G.); mahollin@unmc.edu (M.A.H.)

**Keywords:** RAGE, pancreatic cancer, gemcitabine, cachexia

## Abstract

Pancreatic ductal adenocarcinoma (PDAC) remains a very difficult cancer to treat. Recent in vitro and in vivo studies suggest that the activation of the receptor for advanced glycation end products (RAGE) by its ligands stimulates pancreatic cancer cell proliferation and tumor growth. Additional studies show that, in the RAGE ligand, the high mobility group box 1 (HMGB1) protein plays an important role in chemoresistance against the cytotoxic agent gemcitabine by promoting cell survival through increased autophagy. We hypothesized that blocking the RAGE/HMGB1 interaction would enhance the cytotoxic effect of gemcitabine by reducing cell survival and autophagy. Using a preclinical mouse model of PDAC and a monoclonal antibody (IgG 2A11) as a RAGE inhibitor, we demonstrate that RAGE inhibition concurrent with gemcitabine treatment enhanced the cytotoxic effect of gemcitabine. The combination of IgG 2A11 and gemcitabine resulted in decreased autophagy compared to treatment with gemcitabine combined with control antibodies. Notably, we also observed that RAGE inhibition protected against excessive weight loss during treatment with gemcitabine. Our data suggest that the combination of gemcitabine with a RAGE inhibitor could be a promising therapeutic approach for the treatment of pancreatic cancer and needs to be further investigated.

## 1. Introduction

Pancreatic ductal adenocarcinoma (PDAC) is one of the most lethal cancers [1]. Despite intensive research efforts to explore novel therapies, the cytotoxic drug gemcitabine remains the most widely used for treating PDAC. However, treatment with gemcitabine results in only modest improvements to patient survival and is associated with strong chemoresistance [2,3]. Chemoresistance in PDAC is complex, involving both cellular and molecular mechanisms [4,5,6]. Recent studies suggest that the receptor for advanced glycation end products (RAGE) contributes to chemoresistance in PDAC by promoting cell survival through engagement by the high mobility group box 1 (HMGB1) ligand [7,8]. Blocking RAGE activation by HMGB1 could therefore be an attractive approach to reducing chemoresistance in PDAC. 

RAGE belongs to the large family of immunoglobulin-like cell surface receptors [9]. RAGE is expressed at low levels in most tissues except the lungs, where its expression level is high [10]. RAGE participates in the resolution of inflammation, tissue repair, and bone homeostasis [11,12]. In many diseases, including complications of diabetes, cancer, and Alzheimer’s disease, RAGE is upregulated, and it thus contributes to the progression of these diseases by sustaining an inflammatory milieu [13,14,15,16,17,18]. RAGE is composed of three immunoglobulin-like domains: V-like, C1-, and C2-like domains, a single transmembrane domain, and a short acidic intracellular tail [19]. RAGE is activated by a large number of ligands that bind to the extracellular part of the receptor, mostly the V-domain [20]. RAGE ligands include advanced glycation end products [21], S100 proteins [22], amyloid-forming peptides and proteins [23], HMGB1 [24], the complement C1q protein [25], as well as non-protein ligands such as DNA [26] and glycosaminoglycans [27]. Many RAGE ligands, including HMGB1 and members of the S100 protein family, are associated with tissue damage and inflammatory or metabolic stress and are referred to as damage-associated molecular patterns (DAMPs) [28,29,30]. RAGE is therefore described as a DAMP recognition receptor [19,31]. 

The interaction between RAGE and its ligands results in the activation of several signaling pathways, including the mitogen-activated protein (MAP) kinase pathway, the PI3K/Akt pathway, Jak/STAT (signal transducers and activators of transcription), as well as the Rho GTPases Rac-1 and cdc42 [21,32,33,34,35,36,37,38]. It has been suggested that RAGE is able to activate different pathways by binding to different adaptor proteins on the cytoplasmic domain of the receptor. Four adaptor proteins have been identified so far, including ERK1/2, Diaphanous 1, MyD88, and Toll/interleukin-1 receptor domain-containing adaptor protein (TIRAP). Ultimately, RAGE signaling leads to the activation of several transcription factors, including NF-κB, SP-1, and STAT-3 [39,40,41]. RAGE activation often results in a positive feedback loop because RAGE expression itself is under the control of both NF-κB and SP-1 [41,42,43,44,45,46]. 

Studies have shown that RAGE promotes the formation of pancreatic cancer lesions and tumors. Mice that are knocked-out for RAGE and carry a mutation in oncogenic KRAS (RAGE^−/−^; KRAS^G12D/+^) in their pancreas develop fewer tumors than RAGE^+/+^ mice carrying the same oncogenic KRAS mutation (RAGE^+/+^; KRAS ^G12D/+^) [47,48]. The role of RAGE in promoting pancreatic tumor development is also supported by the observation that RAGE is upregulated in pancreatic lesions and tumors relative to normal adjacent tissues [48]. 

Two RAGE ligands (S100P and HMGB1) play important roles in pancreatic cancer. S100P is a small, calcium-binding protein with important functions in both healthy and neoplastic tissues [49]. The activation of RAGE by S100P in vitro stimulates cell proliferation and migration in human pancreatic cancer cells. In xenograft mouse models of PDAC, RAGE activation by human S100P promotes the growth of pancreatic tumors [50,51,52,53,54,55]. At the cellular level, activation of the RAGE/S100P axis has been shown to promote cell survival against the cytotoxic agents gemcitabine and 5-fluorouracil [50,53], possibly by increasing autophagy and decreasing apoptosis [7]. The second RAGE ligand relevant to pancreatic cancer is HMGB1. HMGB1 is a DNA-binding protein with both intracellular and extracellular functions [56]. As a nuclear protein, HMGB1 regulates gene transcription by binding to and stabilizing DNA. When present in the cytoplasm, HMGB1 regulates autophagy [57]. When secreted into the tumor microenvironment, HMGB1 acts as a damage-associated molecular pattern (DAMP) by interacting with cell surface receptors, including RAGE and the Toll-like receptors (TLR) [17,56,58]. The activation of the RAGE/HMGB1 axis has similar effects on pancreatic cancer cells as the activation of the RAGE/S100P axis [8,59,60]. RAGE activation by HMGB1 has been shown to stimulate pancreatic cancer cell proliferation, migration, and tumor growth, while promoting chemoresistance by sustaining autophagy and decreasing apoptosis [7,40].

Based on these observations, we hypothesized that blocking the interactions of RAGE with its ligands during gemcitabine treatment could reduce tumor cell survival by decreasing autophagy. We tested our hypothesis in an orthotopic mouse model of pancreatic cancer, using a RAGE-specific monoclonal antibody (IgG 2A11) to inhibit RAGE/ligand interactions. We demonstrated that the combination of gemcitabine with IgG 2A11 was more effective at reducing tumor growth than gemcitabine combined with control IgGs. We also showed that RAGE inhibition concurrent to gemcitabine treatment reduced autophagy. Notably, our data further suggested that combining a RAGE inhibitor with gemcitabine treatment could also reduce cancer cachexia. Our study suggests that the addition of a RAGE inhibitor to gemcitabine treatment could be a valuable approach to reduce chemoresistance in pancreatic cancer. 

## 2. Materials and Methods

### 2.1. Cell Culture Conditions

The murine pancreatic cancer cell line KPC #5508 was established from tumors extracted from genetically engineered KPC mice (KrasLSL.G12D/+; p53R172H/+; PdxCretg/+) mice. Cells were grown in DMEM (ATCC, Manassas, VA) supplemented with 10% fetal bovine serum (FBS) (ATCC), penicillin (100 U/mL), and streptomycin (100 µg/mL) (GE Healthcare Life Science, Pittsburg, PA, USA) at 37 °C and 5% CO_2_. The murine melanoma B16F10 cell line and human embryonic kidney (HEK) 293 cells were purchased from ATCC and grown in DMEM supplemented with 10% FBS, penicillin (100 U/mL), and streptomycin (100 µg/mL) at 37 °C and 5% CO_2_. The RAGE transfected HEK293-RAGE cells were a kind gift from Dr. Heizmann (Children’s Hospital, Zurich, Switzerland).

### 2.2. RAGE Quantitative Enzyme-Linked Immunosorbent Assay (ELISA)

A quantitative ELISA (Quantikine ELISA Mouse RAGE Immunoassay kit; R&D Systems, Minneapolis, MN, USA) was performed to determine the level of RAGE protein in the KPC cell line. Three independent cell lysates were prepared from 70–80% confluent cells, detached with a cell scraper, and lysed using a commercial lysis buffer (PARIS Kit, Invitrogen/Thermo Fisher Scientific, Waltham, MA, USA). The total protein concentrations in the cell lysates were determined using the BCA Protein Assay Kit (Pierce/Thermo Fisher Scientific, Walthman, MA, USA). The RAGE protein concentration was determined following the instructions provided by the manufacturer of the ELISA kit.

### 2.3. Cell Viability Assays

KPC #5508 cells were grown to 70–80% confluence and detached with trypsin. Ten thousand cells per well were seeded in 96-well plates and incubated for 24 h. Cells were then treated with gemcitabine concentrations ranging from 0.1 µM to 500 µM and incubated for an additional 48 h. When the effect of RAGE inhibition on the efficacy of gemcitabine was investigated, we used a monoclonal antibody (IgG 2A11) as the RAGE inhibitor. The IgG 2A11 was developed in our laboratory [61]. IgG 2A11 binds to the extracellular part of RAGE and blocks the interaction between the receptor and its extracellular ligands [62]. Changes in cell viability were assessed by measuring differences in Alamar Blue (0.01 mg/mL in each well) fluorescence emission at 590 nm (Ex: 540 nm) after 2 h to 3 h of incubation. The experiment was performed at least three times with six replicates for each condition.

### 2.4. Expression and Purification of IgG 2A11

IgG 2A11 was generated in our lab from a hybridoma cell line and purified from hybridoma supernatants through a single-step protein-G sepharose chromatography [61]. IgG 2A11 binds to the V domain of the extracellular part of RAGE with low nanomolar affinity. IgG 2A11 binds to both the human and murine RAGE extracellular domains. The purity of each batch of the antibody was assessed to be more than 95% by Coomassie blue staining of the SDS polyacrylamide gels. In addition, the binding of each batch of antibody to RAGE was confirmed by surface plasmon resonance using a recombinant for the extracellular domain of RAGE (data not shown).

### 2.5. Generation of the Orthotopic Mouse Model of Pancreatic Cancer

The animal study was performed at the University of Nebraska Medical Center (UNMC) according to the guidelines of the Institutional Animal Care and Use Committees (IACUC) of UNMC and in accordance with the National Institutes of Health Guide for the Care and Use of Laboratory Animals (NIH Publications No. 8023, revised 1978). For the study, 5- to 6-week old male C57Bl/6 mice were purchased from Jackson Laboratories (Bar Harbor, ME) and quarantined for one week to allow them to adapt to the laboratory environment. Mice were implanted with 10,000 KPC #5508 cells in the tails of their pancreases. Mice were allowed to recover from the surgery for five days before starting the treatment.

### 2.6. Mouse Treatment

Mice were enrolled in four groups (*n* = 8 per group) and treated every five days for 30 days. Mice received either a control non-immune isotype antibody (IgG, (NSA mouse IgG (Innovative Research, Novi, MI, USA) (Group I; 100 μg in 100 µL saline); IgG 2A11 (Group II; 100 µg in 100 µL saline); gemcitabine (Group III; 100 mg/kg in 100 µL saline); or the combination of IgG 2A11 (Group IV; 100 µg in 100 µL saline) and gemcitabine (100 mg/kg in 100 µL saline). Saline and gemcitabine solutions were injected intraperitoneally (i.p.), and antibody solutions were injected intravenously (i.v.). At the end of the treatment period, mice were euthanized according to the UNMC IACUC guidelines. At necropsy, the pancreases were collected as quickly as possible. Each pancreas was split into two parts. One half was snap-frozen in liquid nitrogen and stored at −80 °C for Western Blot analysis. The second half was incubated in 10% formalin and later embedded in paraffin for immunohistochemistry (IHC) analysis. The animal study was performed under an approved IACUC protocol at UNMC with protocol number 16-084-08-FC.

### 2.7. Immunohistochemistry

Murine pancreatic tumor tissue slides were sectioned (5 microns) from the paraffin-embedded blocks. Tissues were then stained following standard procedures. Briefly, the slides were first deparaffinized with xylene and rehydrated using a gradient of ethanol and distilled water. An antigen retrieval step was performed in 10 mM sodium citrate buffer, at pH 6, in the presence of 0.05% Tween 20. The slides were treated with 3% H_2_O_2_ for 5 min prior to blocking for 20 min with horse serum at room temperature (RT). Antibody incubation was performed overnight (O/N) at 4 °C for the primary antibody and for 1 h at RT for the secondary antibody. After several washes with TBS-containing 0.05% Tween 20 (TBS-T), the slides were incubated with Vector SG substrate followed by nuclear fast red (Vector Laboratories; Burlingame, CA, USA). A coverslip with permanent mounting media was added to the slides prior to imaging. Images were taken on a Lionheart microscope (BioTek, Winooski, VT, USA).

### 2.8. Western Blots

**Figure 1:**
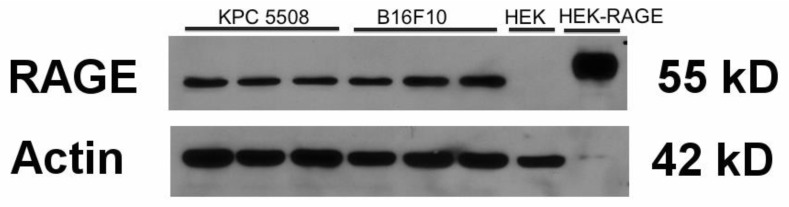
Receptor for advanced glycation end products (RAGE) expression levels in KPC#5508 cells. Three independent cell extracts of KPC#5508 cells and murine B16F10 melanoma cells were used. Extracts of non-RAGE-expressing and RAGE-expressing HEK293 cells were used as negative and positive controls, respectively. Fifty µg protein from each extract was loaded onto the gel, except for RAGE-expressing HEK293 where only 2.5 µg total protein was loaded to avoid signal saturation on the X-ray film.

Protein extracts from the KPC #5508, B16F10, HEK293, and HEK-RAGE cells, as well as from the pancreatic tumors, were prepared using radioimmunoprecipitation assay (RIPA) buffer (Life Technologies, Carlsbad, CA, USA) in the presence of 1 mM phenylmethylsulfonyl fluoride (PMSF) and 1mM of orthovanadate (Thermo Fisher Scientific) to minimize proteolytic degradation and dephosphorylation, respectively. The protein concentration of each cell or tumor extract was determined using the BCA Protein assay kit. For the analysis of the tumor extracts by Western blot, tumor proteins (50 µg to 100 µg) from each treatment group were separated on SDS poly acrylamide gel electrophoresis (PAGE) (10% or 12%, depending on the molecular weight of the protein to be detected) and then transferred onto either nitrocellulose or polyvinylidene difluoride (PVDF) membranes. To avoid oversaturation of the X-ray film, note that only 2.5 µg total protein from HEK-RAGE cell extract was loaded onto the gel performed for Figure 1. Nitrocellulose membranes were used when the blots were developed using X-ray photographic films, and PVDF membranes were used when using the LI-COR CLx Infrared Imaging System (Li-COR Biosciences, Lincoln, NE). Gels contained 4 to 8 samples from each treatment group and were run simultaneously. The nitrocellulose membranes were blocked with 5% non-fat milk powder (*w*/*v*) resuspended in Tris-buffered saline (50 mM Tris-HCl, pH 7.4, 150 mM NaCl, 0.1% Tween 20 (TBS-T)) for 1 h at RT. PVDF membranes were blocked with Odyssey blocking buffer (LI-COR) for 1 h at RT. After blocking, the blots were incubated with the primary antibody (Table 1) O/N at 4°C and finally incubated with the secondary antibody for 1 h at RT.

After blocking, all membranes were incubated simultaneously with the same solution of primary or secondary antibodies, for the same period of time, and washed under similar conditions. The primary and secondary antibodies were diluted either in 5% milk powder in TBS-T (nitrocellulose blots) or Odyssey blocking buffer supplemented with 0.2% Tween-20 (PVDF blots). Peroxidase conjugated secondary antibodies and chemiluminescence substrates (Pierce/Thermo Fisher Scientific) were used when developing the blots with X-ray films. Infrared conjugated secondary antibodies were used when developing the blots with the Odyssey CLx Infrared Imaging System (LI-COR). All blots were developed simultaneously. To confirm that the same amount of protein was loaded into each lane, the blots were incubated with an anti-actin antibody and developed using either an HRP-conjugated antibody (nitrocellulose membranes) or an infrared dye-conjugated secondary antibody (PVDF membrane). Densitometric analysis of the bands from the X-ray films was performed using the ImageJ software [63]. Densitometric analysis of the blots developed using the LI-COR Imager was performed using the software of the LI-COR Imaging System. Western blots were performed at least twice. Representative blots are shown in figures.

### 2.9. Statistical Analysis

Data are presented as means +/− standard deviations. Statistical analysis was performed by using one-way ANOVA. *p*-values of less than 0.05 were considered as statistically significant, and *p*-value cutoffs are indicated as follows: * *p* < 0.05; ** *p* < 0.01; *** *p* < 0.001.

## 3. Results

### 3.1. KPC #5508 Cells Express RAGE

We initiated our study by determining the levels of RAGE in the KPC #5508 cells by Western blot analysis and quantitative ELISA. A single band corresponding to the molecular weight of RAGE (55kD) was observed by Western blot. For comparison, bands of similar size and intensity were observed in cell extracts of murine melanoma B16F10 cells (Figure 1). B16F10 cells have been previously shown to express RAGE [64]. Cell extracts from non-RAGE-expressing and RAGE-expressing HEK293 cells were used as negative and positive controls, respectively. We quantitatively estimated the level of RAGE in the KPC #5508 cells to be 650 pg +/− 70 pg RAGE per mg total protein. Our Western blot analysis and quantitative ELISA data thus show that RAGE was expressed in the murine KPC#5508 cells at a level similar to what is observed in other cancer cell lines, such as the B16F10 murine melanoma cell line. In a recent study, Azizan et al. also reported the expression of RAGE in a distinct KPC cell line generated from (KrasLSL.G12D/+; p53R172H/+; PdxCretg/+) mice [65].

### 3.2. KPC #5508 Cells Are Sensitive to Gemcitabine In Vitro

**Figure 2:**
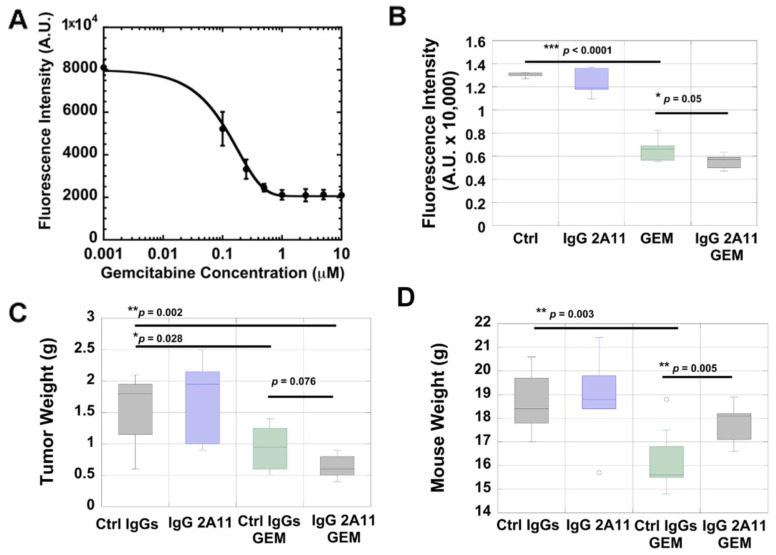
(**A**) Gemcitabine titration in KPC#5508 cells. Fitting of the data revealed an IC_50_ of 100 nM +/− 15 nM. (**B**) RAGE inhibition increases gemcitabine cytotoxicity in KPC #5508 cells, in vitro. Cell viability of KPC #5508 cells was measured after 48 h of treatment with either media alone (Ctrl), 100 nM IgG 2A11, 100 nM gemcitabine, or 100 nM IgG 2A11 and 100 nM gemcitabine. (**C**) RAGE inhibition enhances the cytotoxicity of gemcitabine in vivo. Mice orthotopically implanted with KPC #5508 cells were treated with either non-immune IgGs (Ctrl IgGs), IgG 2A11, Ctrl IgGs and gemcitabine, or the combination of IgG 2A11 and gemcitabine. Pancreatic tumor weights were measured after 3 weeks of treatment. (**D**) RAGE inhibition protects against weight loss in gemcitabine-treated mice. The body weight of the mice was recorded at the end of the three weeks of treatment. * *p* < 0.05; ** *p* < 0.01; *** *p* < 0.001.

We next determined the sensitivity of KPC #5508 cells towards gemcitabine in vitro by measuring cell viability. We observed a gemcitabine dose-dependent decrease in cell viability and calculated an IC_50_ of 100 nM +/− 15 nM on the fitting of the data points (Figure 2A).

### 3.3. RAGE Inhibition Enhances the Cytotoxic Effect of Gemcitabine In Vitro

We investigated if the cytotoxic effect of gemcitabine could be enhanced by RAGE inhibition in vitro. Treatment of the KPC #5508 cells with IgG 2A11 alone did not affect cell viability (Figure 2B). However, when IgG 2A11 was combined with gemcitabine, we observed lower cell viability (*p* = 0.05) than with the treatment of gemcitabine alone (Figure 2B). 

### 3.4. RAGE Inhibition Enhances the Cytotoxic Effect of Gemcitabine in a Preclinical Mouse Model of Pancreatic Cancer

We next investigated the effect of RAGE inhibition on tumor growth in an orthotopic mouse model of melanoma. As expected, we observed that treatment with gemcitabine significantly (*p* = 0.028) reduced tumor growth in mice. Tumors in the gemcitabine group weighed about half as much as those in the control group (Figure 2C). Treatment with the IgG2A11 RAGE antibody alone did not affect the growth of the pancreatic tumors (Figure 2C). However, we observed that IgG 2A11 used in combination with gemcitabine tended to result in smaller tumors than when gemcitabine was combined with control IgGs (*p* = 0.076) (Figure 2C). 

### 3.5. IgG 2A11 Combined with Gemcitabine Results in Reduced Body Weight Loss Compared to Treatment with Gemcitabine and Control IgGs

We observed that the combination treatment of gemcitabine with IgG 2A11 resulted in significantly less bodyweight loss than the treatment with gemcitabine combined with control IgGs (Figure 2D). At the endpoint of the study, the average mouse body weight in the control group was 18.65 g +/− 0.54 g. This weight was reduced in the gemcitabine group (16.2 g +/− 0.48 g), but less reduced in the combination group of gemcitabine with IgG 2A11 (17.8 g +/− 0.28 g), the difference between these last two groups (gemcitabine with control IgGs and gemcitabine with IgG 2A11) was statistically significant (*p* = 0.005) (Figure 2D). 

### 3.6. RAGE Inhibition Reduces Autophagy in Gemcitabine-Treated Tumors

**Figure 3:**
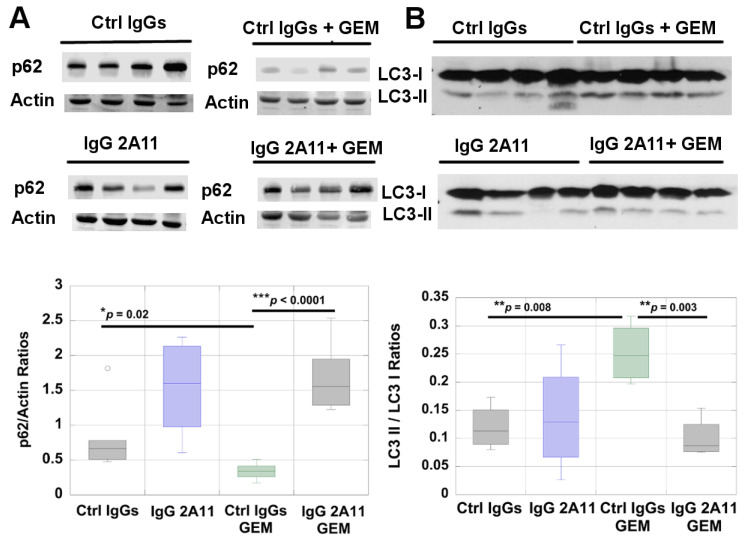
RAGE inhibition reduces autophagy in gemcitabine-treated tumors. Autophagy was assessed by measuring (**A**) the levels of p62 and (**B**) the levels of LC3-I and LC3-II, in tumor extracts from the different treatment groups by Western blot analysis (*n* = 4 in each group). The calculated ratios of LC3-II/LC3-I have been plotted. Representative blots are shown in A and B. * *p* < 0.05; ** *p* < 0.01; *** *p* < 0.001.

We determined if autophagy in pancreatic tumors was affected by RAGE inhibition. Autophagy is a dynamic process that involves the formation and degradation of autophagosomes, where organelles and proteins are degraded or recycled [66]. We used two complementary approaches to assess autophagic activity in the tumor extracts: we measured the levels of the p62 protein and we calculated the ratios of the two forms of the microtubule-associated protein light chain 3 (LC3-I and LC-3-II) in the different treatment groups. The p62 protein, also named sequestosome 1 (SQSTM1), serves as an adaptor protein that links ubiquitinated proteins to the autophagic machinery and enables their clearance in the phagolysosomes [67]. The p62 protein is also degraded during autophagy, and accumulation of p62 indicates inhibition of autophagy while a decrease in p62 levels indicates increased autophagy [67]. Western blot analysis of p62 in the different treatment groups revealed significantly lower levels of p62 in the gemcitabine-treated tumors than in the control tumors, suggesting that the gemcitabine treatment increased autophagy (Figure 3A). Higher levels of p62 were also observed in mice treated with IgG2A11 alone or with the combination of IgG2A11 and gemcitabine, suggesting that RAGE inhibition reduced autophagy in these tumors (Figure 3A). 

We next determined if RAGE inhibition also affected the expression levels of LC3-I and LC3-II by Western blot analysis and by calculating LC3-II/LC3-I ratios in the different tumor extracts [68] (Figure 3B). The cytosolic form of LC3 (LC3-I) becomes conjugated with phosphatidylethanolamine during autophagy and forms the LC3-II form, which is associated with autophagosome membranes. The quantity of LC3-II relates to the number of autophagosomes that are formed, and the ratio of LC3-II/LC3-I correlates with active autophagy [68]. However, because autophagy is a dynamic process, LC3 II may not always be detected during autophagy because of the active degradation of autophagosomes. 

We observed significantly (*p* = 0.008) higher ratios of LC3-II/LC3-I in the gemcitabine-treated tumors than in the tumors treated with the control IgGs, suggesting an increase in autophagosome formation and thereby autophagic activity (Figure 3B). Although mice treated with IgG 2A11 alone did not change their ratios of LC3-II/LC3-I in tumor extracts, the treatment with IgG 2A11 combined with gemcitabine resulted in significantly lower LC3-II/LC3-I ratios than the treatment with gemcitabine combined with the control IgGs (Figure 3B). Collectively, our Western blot analysis of p62 and LC3-I/LC3-II levels in the tumor extracts suggest that RAGE inhibition reduced gemcitabine-induced autophagy in these pancreatic tumors.

### 3.7. RAGE Inhibition Increases Poly(ADP-Ribose)Polymerase (PARP) Activation in Tumors Treated with Gemcitabine

**Figure 4:**
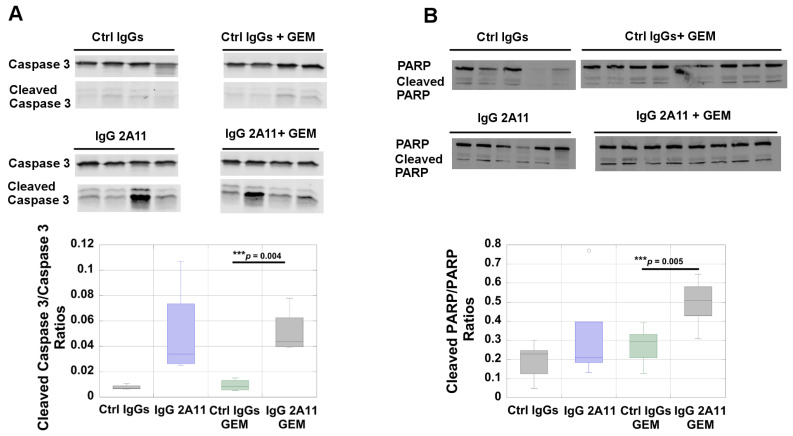
RAGE inhibition increases apoptosis in gemcitabine-treated tumors. Apoptosis was assessed by measuring the ratios of (**A**) cleaved caspase 3/caspase 3 and (**B**) cleaved PARP/PARP in different tumor extracts (*n* = 4 to 8 per treatment group). *** *p* < 0.001.

We next investigated if RAGE inhibition affected the levels of apoptosis in tumors. Apoptosis, or programmed cell death, is often triggered by excessive DNA damage that cannot be properly repaired, such as that resulting from gemcitabine treatment [69]. The exposure of pancreatic cancer cells to gemcitabine generally leads to the stimulation of apoptosis. However, resistance to apoptosis has been observed in many human pancreatic cancer tumors and multiple types of resistance mechanisms have been described [69]. Apoptosis can be initiated by the effector caspases, including caspases 3, 7, and 9 [70,71]. Another effector of apoptosis is the poly(ADP-ribose)polymerase (PARP). During apoptosis, PARP is cleaved, and PARP cleavage has become a useful marker of this type of cell death [72]. We analyzed the levels of caspase 3 and cleaved caspase 3 in the different tumor extracts by Western blot analysis, and observed significantly higher levels (*p* = 0.004) of cleaved caspase 3 and caspase 3 in the tumors treated with IgG 2A11 and gemcitabine than in the tumors treated with control IgGs and gemcitabine (Figure 4A). Similarly, an analysis of PARP levels by Western blot revealed significant differences (*p* = 0.005) in the cleaved PARP (89 kd)/PARP ratios between the tumors treated with gemcitabine and the control IgGs and the tumors treated with gemcitabine and IgG 2A11, the highest ratios observed were with the combination treatment of IgG 2A11 and gemcitabine (Figure 4B).

### 3.8. RAGE Inhibition Reverses Gemcitabine-Dependent Increases in ERK1/2 Activation

The extracellular signal-related kinases 1 and 2 (ERK1/2) are two kinases that transmit mostly mitogenic signals from the receptor tyrosine kinases, G protein-coupled receptors, ion channels, and other types of receptors [73]. ERK1/2 are typically activated (phosphorylated) in response to the activation of the small Ras GTPase, and the RAF and MAP kinases [74,75,76]. An ERK1/2 cascade can also be stimulated in response to RAGE activation by its ligands [41,65]. In particular, Azizan et al. recently reported that treatment of mice that had been orthotopically implanted with KPC cells with the small-molecule FPS-ZM1 RAGE inhibitor showed reduced levels of phosphorylated ERK1/2 in their tumors than did the control tumors. These authors concluded that RAGE signaling strongly contributed to ERK activation in their mouse model of pancreatic cancer [65]. In addition, recent studies have shown that ERK1/2 activation can also reflect some degree of chemoresistance [75,77]. For instance, increased levels of phosphorylated ERK1/2 were observed in a panel of three human pancreatic cancer cell lines treated with gemcitabine [78]. Another study showed that phosphorylated ERK1/2 protected melanoma cells from undergoing cis-platin-mediated apoptosis [79]. Based on these observations, we investigated if RAGE inhibition and gemcitabine treatment affected the levels of ERK1/2 in the tumors from the different treatment groups. 

**Figure 5:**
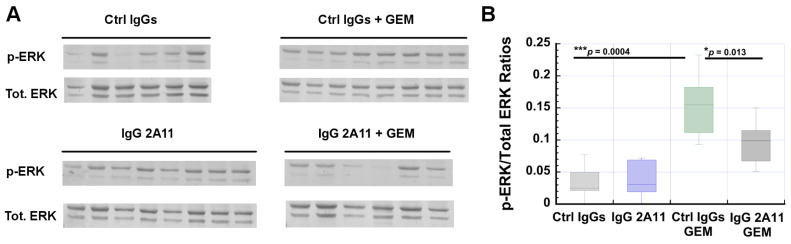
RAGE inhibition reduces gemcitabine-mediated increases of pERK1/2. (**A**) Western blots were performed with six to eight tumor extracts from each treatment group. Representative blots are shown. (**B**) Densitometric analysis of the blots. * *p* < 0.05; *** *p* < 0.001.

We determined the levels of phosphorylated ERK1/2 (p-ERK) (at Threonine 202 and/or Tyrosine 204 in ERK1 and Threonine 185 and/or Tyrosine 187 in ERK2) in the tumor extracts from all treatment groups, and found significantly higher levels of p-ERK in the tumors treated with gemcitabine than in the control tumors (*p* = 0.0004) (Figure 5).

Importantly, we observed a significant reduction in p-ERK levels (*p* = 0.013) when IgG 2A11 was combined with gemcitabine. The treatment of mice with IgG 2A11 alone did not affect the levels of p-ERK in the tumors, compared to the tumors treated with the control IgGs. Our data thus suggest that RAGE inhibition reduces gemcitabine-mediated increases in ERK1/2 activity.

### 3.9. RAGE Inhibition Reverses Gemcitabine Mediated Increases in NF-κB Activation

The transcription factor “nuclear factor-κB” (NF-κB) plays a critical role in inflammation, immunity, cell proliferation, and apoptosis [80]. Studies have shown that NF-κB also has important functions in pancreatic cancer, and is found constitutively activated in the tumors of many pancreatic cancer patients [81]. 

**Figure 6:**
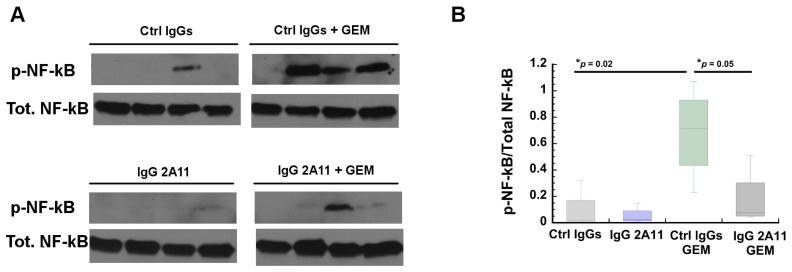
RAGE inhibition reduces gemcitabine-mediated increase in NF-κB activation. (**A**) NF-κB activation was determined by measuring the levels of p-p65 by Western blot analysis. Western blots were performed with four tumor extracts from each group. Treatment of mice with IgG 2A11 alone did not affect the levels of p-p65 in tumors as compared to tumors treated with the control IgGs. Our data thus suggest that RAGE inhibition reduces gemcitabine-mediated increases in NF-κB activity. (**B**) Densitometric analysis of the blots. * *p* < 0.05.

Importantly, NF-κB is activated following the activation of RAGE by many of its ligands, including HMGB1 [17,41,82]. In the classical pathway, NF-κB activation involves the phosphorylation of IκBα by the activated IKKα/IKKβ kinase. Following its phosphorylation, IκBα dissociates from the heterocomplex formed with the p65/p50 heterodimer, and p65/p50 can then translocate to the nucleus and act as an activator of transcription [80]. Phosphorylated IκBα is then ubiquitinated and sent for proteasomal degradation [80]. In many instances, the transcriptional activity of the p65/p50 heterodimer is enhanced by phosphorylation on the p65 subunit [83]. To determine if RAGE inhibition modulated the activation of the classical NF-κB pathway, we compared the levels of phosphorylated p65 between the tumors from the different treatment groups. We observed statistically significant (*p* = 0.02) higher levels of phosphorylated p65 (p-p65) in the tumors treated with gemcitabine and control IgGs than in the tumors treated with control IgGs only. However, in the presence of RAGE inhibitors, we observed that the levels of p-p65 were statistically significantly reduced (*p* = 0.05), suggesting that the increase in NF-κb activity in the gemcitabine treated cells was in part caused by activation of the RAGE signaling pathway (Figure 6).

### 3.10. RAGE and HMGB1 Are Expressed in Murine Tumor Tissues

**Figure 7:**
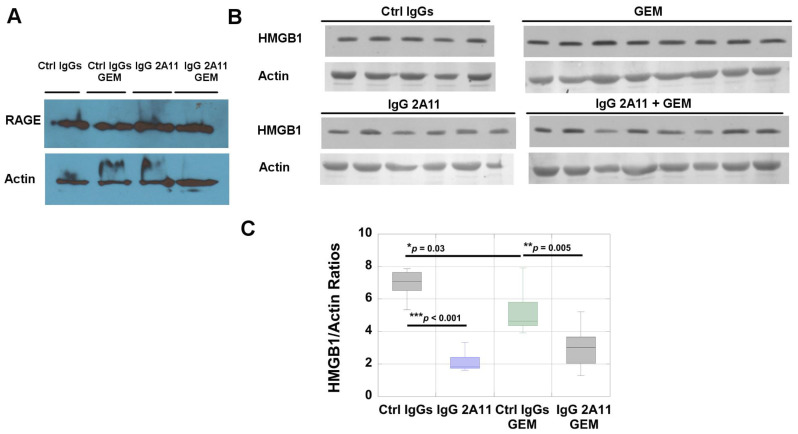
(**A**) RAGE expression in representative tumor extracts from each treatment group. (**B**) HMGB1 expression in tumor extracts. (**C**) Densitometric analysis of the blot shows a significant decrease in HMGB1 levels upon RAGE inhibition in gemcitabine-treated tumors. * *p* < 0.05; ** *p* < 0.01; *** *p* < 0.001.

Western blot analysis revealed that RAGE was expressed in tumor extracts from each treatment group (Figure 7A). 

Western blot analysis also revealed the presence of HMGB1 in tumors from all treatment groups (Figure 6B). We observed significantly lower levels of HMGB1 in tumor extracts from the mice treated with gemcitabine and the control IgGs than in the mice treated with control IgGs alone (*p* = 0.03). Similarly, treatment with IgG 2A11 resulted in lower levels of HMGB1 in the tumor extracts of the mice treated with IgG 2A11 than in the mice treated with the control IgGs (*p* < 0.001). More importantly, we observed that RAGE inhibition was associated with lower levels of HMGB1 in the tumor extracts treated with gemcitabine (*p* = 0.005) than in the mice treated with gemcitabine and the control IgGs. 

**Figure 8:**
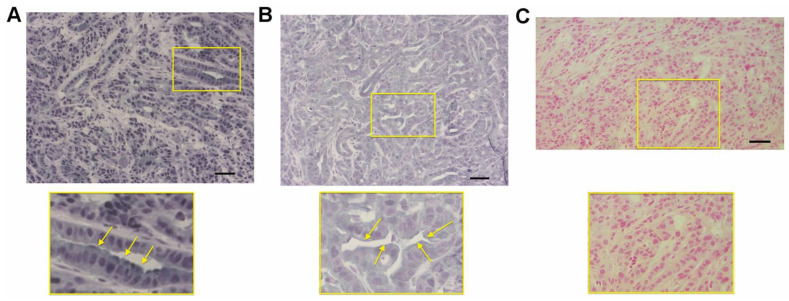
Immunohistochemical analysis of murine pancreatic tumors generated from KPC #5508 cells. (**A**) Staining for HMGB1. (**B**) Staining for RAGE. (**C**) Control staining where the primary antibody was omitted. In (**A**–**C**), the regions defined in the yellow rectangles are shown at higher magnification. Arrows indicate (**A**) staining for HMGB1 in the luminal part of the cells and (**B**) cell surface staining for RAGE. All slides come from the same tumor block. Scale bar: 50 µm.

The presence of RAGE was also revealed by immunohistochemistry in the tumor sections. Positive RAGE staining was observed in the cytoplasm and at the cell membrane (Figure 8). HMGB1 was also detected in the KPC #5508 murine tumors, where nuclear and cytoplasmic staining were observed (Figure 8). The presence of S100P was not investigated because S100P is not a functional protein in mice.

## 4. Discussion

This is the first study to demonstrate the effect of RAGE inhibition on the efficacy of gemcitabine in a preclinical mouse model of pancreatic cancer. We used a mouse model that is clinically relevant to human pancreatic ductal adenocarcinoma [84,85]. Indeed, in our model, murine KPC cells were implanted directly in the pancreas of the mice. The KPC #5508 cell line was established from pancreatic tumors that had been developed in genetically engineered Kras(G12D), Trp53(R172H), and Pdx1-Cre (KPC) mice. We used murine and not human cancer cells for the mouse model because this allowed us to use mice (C57Bl/6 mice) with a competent immune system. Similar models have been used by other investigators and were found to have high translational potential for the study of therapeutic drugs [84,85]. In our study, we showed that the KPC #5508 cell line expressed RAGE (Figure 1) and was sensitive to gemcitabine (Figure 2A). In a different study, Azizan et al. had also shown that tumors generated from a different KPC cell line expressed RAGE [65]. We showed in this study that RAGE inhibition enhanced the cytotoxicity of gemcitabine both in vitro and in vivo. In the mouse model, we observed that the difference in tumor weight averages between the combination groups of gemcitabine/control IgGs and gemcitabine/IgG 2A11 showed 92.4% confidence (*p* = 0.076), which is close to the 95% confidence interval (*p* = 0.05) generally used to define statistical significance. In both in vitro and in vivo conditions, we showed that treatment with IgG 2A11 alone did not impact the growth of tumors. These data support the hypothesis that RAGE inhibition is effective only when RAGE ligands are released into the tumor microenvironment. The two RAGE ligands S100P and HMGB1 have been shown to stimulate RAGE in human pancreatic cancer cells and tumors and to promote pancreatic tumorigenesis. However, in our animal model, only HMGB1 was a relevant ligand because mice do not express functional S100P. We suggest that IgG 2A11 effectively enhances the cytotoxic effect of gemcitabine by blocking RAGE interaction with extracellular HMGB1. In support of our hypothesis, we detected RAGE and HMGB1 in tumors from all treatment groups by both immunohistochemistry and Western blot analysis. 

HMGB1 is a complex molecule with nuclear, cytoplasmic, and extracellular functions. A comparison of the levels of HMGB1 in the tumor extracts by Western blot analysis (Figure 7) revealed differences between the different treatment groups. Of importance, we observed that treatment of mice with IgG 2A11 resulted in reduced levels of HMGB1 in tumors compared to the treatment of mice with the control IgGs. Similarly, the combination of IgG 2A11 with gemcitabine resulted in lower levels of HMGB1 in tumors than the combination of the control IgGs with gemcitabine, suggesting that RAGE inhibition might modulate HMGB1 levels, either by reducing the expression of HMGB1 in tumor tissues or by increasing the secretion of HMGB1 extracellularly. Additional experiments would be needed to answer this important question. 

Our data further suggested that RAGE inhibition reduced gemcitabine-induced autophagy, as illustrated by the increased p62 levels and by the decrease in the ratios of LC3-II/LC3-I in the gemcitabine/IgG 2A11 combination group, as compared to the gemcitabine/control IgGs group. Autophagy is a complex intracellular catabolic process that is involved in both the promotion of tumor formation and tumor inhibition [86,87]. Previous studies have shown that the HMGB1/RAGE axis promoted autophagy in pancreatic tumors [7]. Our data are in agreement with this study, as we showed that RAGE inhibition modulated both autophagy and HMGB1 levels in tumors. However, additional experiments are necessary to determine if HMGB1 promotes autophagy through its interaction with RAGE in our experimental model of pancreatic cancer. In addition to affecting autophagy, we observed that RAGE inhibition resulted in increased apoptosis in the tumors treated with gemcitabine (Figure 4). Autophagy and apoptosis are multifaceted and very complex processes, and the possibility of communication between these processes has been the topic of many reviews over the last few years [88]. Additional experiments would be needed to elucidate possible cross-talk between autophagy and apoptosis in our experimental conditions.

Interestingly, we observed increased ERK1/2 activity in the tumors treated with gemcitabine (Figure 5). ERK1/2 are kinases that are involved in a large number of cellular processes and, in cancer tumors, they frequently transmit mitogenic signals [73]. A recent report suggested that in pancreatic cancer cells, ERK1/2 activity contributes to gemcitabine resistance [78]. ERK1/2 activity can also increase as a result of RAGE activation by its ligands [16,41]. Indeed, the recent study of Azizan et al. showed that RAGE inhibition with the small molecule RAGE inhibitor FPS-ZM1 was sufficient to reduce ERK1/2 activity in tumors from an orthotopic mouse model of pancreatic cancer [65]. This observation suggests that in our study, the decrease in phosphorylated ERK1/2 levels detected in mice treated with IgG 2A11 and gemcitabine could thus be caused by RAGE inhibition. 

We also observed that RAGE inhibition affected the classical NF-κB signaling pathway by decreasing the phosphorylation levels of p65, further supporting the hypothesis that the RAGE signaling pathway contributes to tumor survival in the presence of gemcitabine, and that RAGE inhibition could enhance the effect of gemcitabine in tumors (Figure 6). In pancreatic cancer, multiple signaling pathways have been shown to cross-talk with the NF-kB signaling pathway, resulting in tumorigenesis, tumor survival, metastasis, or chemoresistance [89]. These pathways include, among others, the KRAS pathway, the TGF-β pathway, and the Notch pathways [90,91,92,93,94]. Recently, Azizan et al. showed that activation of the RAGE signaling pathway also contributed to NF-κB signaling in human (Panc-1) and murine (KPC) pancreatic cancer cells [65]. In these cells, RAGE inhibition using either the small molecule FPS-ZM1 or an anti-RAGE antibody was found to reduce the basal levels of NF-κB, as shown by reduced levels of phosphorylated phosphor-IKba [65]. Although we did not observe significant changes in the basal levels of NF-κB in the KPC murine tumors, we showed that the RAGE signaling pathway contributed to NF-κB signaling in the presence of gemcitabine (Figure 6).

Finally, our observation that mice treated with gemcitabine and IgG 2A11 lost significantly less weight than mice treated with gemcitabine with the control IgGs (Figure 1C) is in agreement with a recent study by Chiappalupi et al. [95]. Using RAGE knock-out mice, Chiappalupi et al.demonstrated that RAGE signaling was a determinant factor in muscle protein degradation, pro-inflammatory cytokines, and tumor-derived cachexia-inducing factors in colon carcinoma and Lewis lung carcinoma models of cancer [95]. The reduced weight loss observed in the gemcitabine/IgG 2A11 combination group could thus reflect reduced cancer cachexia in these mice.

## 5. Conclusions

In conclusion, our data present evidence that RAGE inhibition can enhance the toxicity of gemcitabine in a preclinical mouse model of pancreatic cancer. Our data further support the role of RAGE signaling in increasing autophagy and possibly decreasing apoptosis in pancreatic tumors. Our data also suggest a role for RAGE signaling in cancer cachexia. Collectively, our data suggest that the combination of a RAGE inhibitor with gemcitabine could be a valuable approach to improving pancreatic cancer treatment and reducing cancer-associated cachexia.

## Figures and Tables

**Table 1:** Antibodies used in this study.

Antigen	Provider/Catalog Number	Application	Dilution
P62 (SQSTM1)	Santa Cruz Biotechnologies/28359	WB	1/1000
LC3A/B	Cell Signaling Technology/12741	WB	1/500
Cleaved caspase 3	Cell Signaling Technology/9662	WB	1/1000
Caspase 3	Cell Signaling Technology/9661	WB	1/1000
PARP	Cell Signaling Technology/9542	WB	1/1000
HMGB1	Cell Signaling Technology/3935	IHC	1/1000
HMGB1	Thermo Fisher Scientific/PA5-27378	WB	1/100
Phospho-ERK1/2	Cell Signaling Technology/9101	WB	1/1000
ERK1/2	Cell Signaling Technology/4695	WB	1/1000
Phospho-NF-KB p65	Cell signaling Technology/3033	WB	1/1000
Total NF-kB p65	Cell Signaling Technology/8242	WB	1/1000
RAGE	Thermo Fisher Scientific/PA1-075	WB	1/200
RAGE	R&D Systems/AF1145	IHC	1/1000
β-actin	Cell Signaling Technology/4967	WB	1/1000
HRP-donkey anti-rabbit	Jackson ImmunoResearch/711-035-152	WB	1/25,000
IRdye800 goat anti-rabbit	LI-COR/P/N 927-50000	WB	1/50,000
IRdye680 goat anti-rabbit	LI-COR/P/N 926-68071	WB	1/50,000

## Data Availability

Data is contained within this article.
